# Inequalities in measles immunization coverage in Ethiopia: a cross-sectional analysis of demographic and health surveys 2000–2016

**DOI:** 10.1186/s12879-020-05201-5

**Published:** 2020-07-07

**Authors:** Gebretsadik Shibre, Betregiorgis Zegeye, Dina Idriss-Wheeler, Sanni Yaya

**Affiliations:** 1 Department of Reproductive, Family and Population Health, School of Public Health, Addis Ababa University, Addis Ababa, Ethiopia; 2 Shewarobit Field Office, HaSET Maternal and Child Health Research Program, Addis Ababa, Ethiopia; 3 Interdisciplinary School of Health Sciences, University of Ottawa, Ottawa, Canada; 4 School of International Development and Global Studies, University of Ottawa, Ottawa, Canada; 5 The George Institute for Global Health, The University of Oxford, Oxford, UK

**Keywords:** Measles immunization, Health disparities, Child health, Global health, Demographic and health surveys, Ethiopia

## Abstract

**Background:**

Ethiopia has low measles immunization coverage and little is known about the disparities surrounding what coverage is provided. This study assessed disparities in measles immunization and its change over time using the four Ethiopia Demographic and Health Surveys conducted between 2000 and 2016.

**Methods:**

This is a cross-sectional analysis of data using Ethiopia Demographic and Health Surveys (EDHS) conducted between 2000 and 2016. We used the World Health Organization’s (WHO) Health Equity Assessment Toolkit (HEAT) to present the inequalities. Four measures of inequality were calculated: Difference (D), Ratio (R), Population Attributable Fraction (PAF) and Population Attributable Risk (PAR). The results were disaggregated by wealth, education, residence, sex and sub-national regions and 95% Uncertainty Intervals (UIs) were computed for each point estimate to boost confidence of the findings.

**Results:**

Measles immunization coverage was higher among the richest and secondary and above schools’ subgroup by nearly 30 to 31 percentage points based on point estimates (D = 31%; 95% CI; 19.48, 42.66) and 29.8 percentage points (D = 29.8%; 95% CI; 16.57, 43.06) as compared to the poorest and no education subgroup respectively in the 2016 survey. Still, in the 2016 survey, substantial economic status (PAF = 36.73; 95%CI: 29.78, 43.68), (*R* = 1.71; 95%CI: 1.35, 2.08), education status (PAF = 45.07; 95% CI: 41.95, 48.18), (*R* = 1.60; 95% CI: 1.30, 1.90), place of residence (PAF = 39.84, 95% CI: 38.40, 41.27), (*R* = 1.47, 95% CI: 1.20, 1. 74) and regional (PAF = 71.35, 95% CI: 31.76, 110.95), (*R* = 3.09, 95%CI: 2.01, 4.17) inequality were observed with both simple and complex measures. There was no statistically significant difference in the prevalence of measles immunization between male and female children in all the studied years, as indicated, for instance, by measures of PAF in 2000 (PAF = 0; 95%CI: − 6.79, 6.79), 2005 (PAF = 0; 95%CI: − 6.04, 6.04), 2011(PAF = 0; 95%CI: − 3.79, 3.79) and 2016 (PAF = 2.66; − 1.67; 6.99). Overall, the inequality of measles immunization narrowed significantly by at least some of the measures between the first and the last survey periods across all the studied subgroups.

**Conclusions:**

National, regional and district levels of government should make a pledge to reduce inequalities in coverage of measles immunization. Equity-sensitive strategies, sufficient human and financial resources as well as continued research and monitoring of immunization coverage inequalities are necessary to achieve related sustainable development goals.

## Background

Measles is a highly infectious disease, causing epidemics in high income countries and is a leader in the mortality among children in low income countries [1, 2]. In 2014, measles led to 114, 900 deaths globally, 63% (73,914) of which were in Africa [3, 4]; it is the main cause of child mortality in sub-Saharan Africa [5]. There has been progress in the reduction of measles mortality in the last decade in Africa [6], however, the disease remains an issue in the region [5, 7, 8], reflecting the challenge of sufficient herd immunity levels in areas where financial resources are limited.

Childhood immunizations are low-cost, safe and effective interventions for reducing infant and under-five morbidity and mortality [9]. The majority (90%) of the unimmunized population are in low and middles income countries, many of whom are in South-East Asia and Africa [10]. In 2015, 20.8 million infants did not receive the first dose of the Measles Containing Vaccine (MCV1) through routine immunization; Ethiopia accounted for 3.4% of unimmunized infants [11] and 9% of the global measles mortality [12, 13].

Ethiopia’s childhood immunization coverage has improved through a combination of strategies that include the Reaching Every District (RED) approach, health extension programs and implementation of Enhanced Routine Immunization Activities (ERIAs) [14–16]. With the increased measles immunization approach, the incidence rate of measles was expected to decrease [17]. Despite improvement of childhood vaccinations, measles outbreaks continue in most of Ethiopia. Even though MCV1 coverage increased from 59% in 2005 to 84% in 2014 [14, 15, 18], the incidence rate of confirmed measles cases per 100,000 increased from 0.6 in 2005 to 11.2 in 2014 [15]. The persistent occurrences of measles outbreaks despite vaccination is thought to be attributed to spatial heterogeneity of measles vaccination [15, 19].

Vaccine coverage can vary due to geographical, socioeconomic or demographic factors; therefore, inequalities of coverage can emerge based on household economic status, mother’s education, and place of residence [20]. The *2016 WHO State of Inequality: Childhood Immunization* report demonstrated a 20 percentage point difference between the most and least educated sub-groups, suggesting that high levels of education-related absolute inequality continue despite gains in national coverage in Ethiopia [20].

National immunization coverage rates tend to mask disparities among sub-populations within a country. To understand who the unimmunized children are, it is important to not only investigate the national averages, but also look at the distribution of immunization within a country’s sub-groups [21]. Inequalities by household wealth, mother’s education and urban or rural residence are common in childhood immunization [21].

Continued immunization coverage without focus on inequalities will not address gaps in coverage. It is important to have an equity-focused approach that concentrates on barriers to supply and demand of immunization. This reinforces that the hardest to reach populations are at the center of the expanded coverage strategies while also sustaining the gains made to date [22, 23].

Not only must a country achieve high national coverage, it must also reach the children who remain unimmunized [21]. In addition to the moral imperative of equitable coverage, there are economic and cost-effective advantages to addressing inequalities in immunization coverage [21, 24].

Assessing the state of inequality in immunization identifies gaps and informs planning strategies regarding increased coverage in unvaccinated or under-vaccinated population subgroups [20]. A few studies in Ethiopia focused on socio-economic inequalities in immunization using different dimensions of inequality and summary measures [25, 26]. This study expanded on previous studies and assessed socio-economic inequality using five dimensions of inequality; economic status, educational status, place of residence, sex and sub national regions. Furthermore, inequality was assessed using simple, complex, relative and absolute measures. Finally, it filled the gap of evidence in trends of inequality. The main aim of the study was to assess prevalence and trends of inequality in childhood measles immunization in Ethiopia from 2000 to 2016.

## Methods

### Study setting

Ethiopia is the second most populous country in Africa with a population of 109 million [27].The country experienced strong economic growth over the last decade, and the agricultural sector has been the main driver of the growth [27]. Despite the decline in its share of the population living in poverty [27], Ethiopia continues to be one of the poorest nations in the world with a 2018 estimated average per capita Gross Domestic Product (GDP) of US $ 772.3 [28]. Furthermore, it was ranked 174 out of 188 countries in the 2016 Human Development Index (HDI) report, indicating the country’s poor performance in the dimensions of education, health and income used to create the index [29].

Globally, Ethiopia has a high burden of maternal and neonatal mortality despite improvements over the past 20 years [30–32]. Mothers continue to die from pregnancy related complications even though there have been increases in overall coverage of maternal health services in the country [33]. These could potentially be due to the inequitable distribution of the services favoring advantaged subpopulations [34]. Ethiopia adopted a Health Sector Transformation Plan (HSTP) in 2015 to be implemented over 5 years [35]. The plan aims to improve maternal and child health by focusing on equitable distribution of health care services.

### Data sources

Cross-sectional data from four Ethiopia Demographic and Health Surveys (DHSs), between 2000 and 2016, were used. A two-stage cluster design was employed with 28 to 30 households selected in the second stage for sampling. The nationally representative DHS survey collected information on several public health related topics such as anthropometric, demographic, socioeconomic, family planning and domestic violence. They were implemented in Ethiopia with the financial and technical assistance by Inner City Fund (ICF) International provisioned through the USAID-funded MEASURE DHS program.

### Selection of variables

Measles immunization coverage was computed for 1-year-old children. It is the percentage of children aged 12–23 months who received at least one dose of the measles vaccine before the survey according to a vaccination card (shown to the interviewer), the mother’s report, or health facility visit (this source is only available for 2016 EDHS). When no card was shown to the interviewer (i.e. this option was missing), the inofrmation on whether or not the child was vaccinated for measles came from the mother's report. When the mother’s report for vaccination was missing, then this was coded as no measles vaccine was given. Economic status was proxied through a wealth index in the DHS computed using household assets and ownership following the methodology explained elsewhere [36] and was classified as poorest, poor, middle, rich and richest. The wealth index was computed for each of the four surveys conducted in Ethiopia using principal component analysis (PCA) and deemed comparable across the survey years. Maternal education status was classified as noeducation, primary education, and secondary education or higher, place of residence as urban vs. rural, and the sub-national region included nine regions and two city administrations.

### Data analysis

We used the WHO’s HEAT version 3.1 (2019 update) software for the analysis [37]. Re-analysis of the DHS datasets were completed by the WHO and stored in the WHO Health Equity Monitor database [38]. The database considered the survey design specifications during analysis to produce findings that were not biased.

The HEAT software makes use of the stored data and allows researchers to complete inequality analysis of health indicators such as measles immunization. In this study, inequality in the prevalence of measles immunizations (i.e. measles immunization coverage) was disaggregated by five equity stratifiers (economic status, education, place of residence, region, and sex) and analyzed using four commonly used summary measures of health inequality [39]: Difference (D), Ratio (R), Population Attributable Risk (PAR) and Population Attributable Fraction (PAF). Simple and complex, as well as relative and absolute summary measures were calculated for the equity stratifiers to investigate inequality in the coverage of measles immunization among sub-groups. Using different summary measures in an inequality study has been recommended by the WHO to produce policy-relevant findings [38]. The Difference and Ratio are simple measures whereas PAR and PAF are complex measures [37, 39]. The simple measures of health inequality are useful to compare indicators between just two subgroups and do not use the entire subpopulations of a variable to produce values. Complex measures, on the other hand, take into account the size of the entire subgroup of a variable which overcome this limitation inherent to simple measures [39].

Detailed account of the summary measures can be found elsewhere [37, 39]. But briefly, D was calculated as measles immunization in the richest group minus the poorest group, at least secondary education or higher minus the no education group, and urban minus rural populations. For the sub-national regions, the D was calculated as the difference between region with the highest estimate and the region with lowest estimate for measles immunization coverage. For sex, measles immunization for female minus that of male was done. Ratio is calculated as the ratio of two subgroups: *R* = Y_high_ / Y_low_. For place of residents, Y_high_ and Y_low_ are urban and rural residents respectively. Whereas in educational status, Y_high_ and Y_low_ refers to the most advantaged subgroups (secondary schools or higher) and the most disadvantaged subgroups (no education). For economic status, Y_high_ and Y_low_ refers to the richest quintile and the poorest quintile, respectively. Finally, for sex, the calculation was the ratio of female children measles immunization (Y_high_) to male children measles immunization (Y_low_).

PAR is calculated as the difference between the estimate for the reference subgroup (yref) and the national average of measles coverage (μ): PAR = yref-μ, where is μ national average of measles coverage. In this study, yref refers to estimates of measles immunization in the: (i) urban setting for place of residence, secondary education for education, and the richest sub-groups for economic-based differences. For the dimension of inequality at the sub-national regional level, yref refers to the sub-national region with the highest estimate of measles immunization coverage. PAF is the relative inequality dimension of the PAR and is calculated through the formula: PAF = (PAR/ μ)*100. Zero for PAR and PAF indicates absence of inequality and the greater absolute value indicates a higher level of inequality.

As a measure of statistical significance, 95% Uncertainty Intervals (UI) were computed around point estimates. To state that significant inequality existed, the lower and upper bounds of Difference and PAR UIs should not include zero. R and PAF inequality exist if UIs do not include one. To demonstrate trend over time inequality, UIs of the summary measures between the survey years must not overlap. This study followed the Strengthening the Reporting of Observational Studies in Epidemiology (STROBE) guidelines [40].

### Ethical consideration

The study used publicly available data from the Ethiopia Demographic and Health Surveys. Ethical considerations were the responsibly of and completed by the institutions that commissioned, funded, and managed the surveys. All DHS surveys are approved by ICF international as well as an Institutional Review Board in the respective country to ensure that protocols are in compliance with the U.S. Department of Health and Human Services regulations for the protection of human subjects.

## Results

### Extent and trends of measles immunization across different dimensions of inequality

**Fig. 1:**
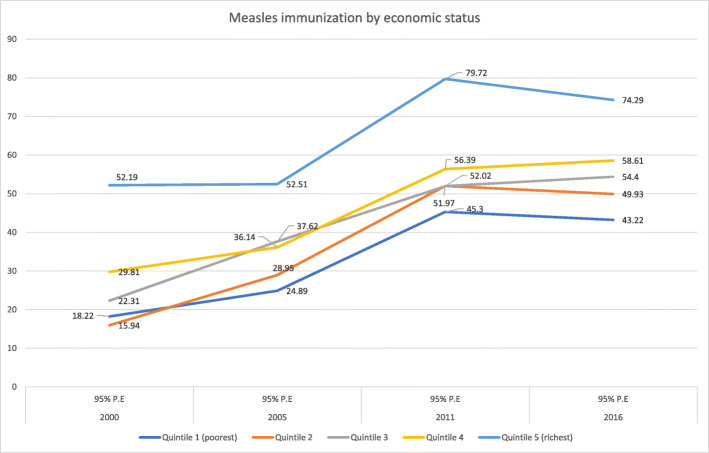
Coverage and trends of childhood measles immunization based on economic status in Ethiopia from 2000 to 2016

The study included a combined total of 7, 951 participants from the four demographic and health surveys. Close to 50% were female (3949), 70% (5536) resided in rural regions, 72% (5725) were not educated, and 23.2% (1852) were from the poorest wealth quintile. The national coverage of measles immunization increased from 2000 (27%) to 2005 (35%), then again in 2011 (56%) but leveled off by 2016 (54%). Table 1 illustrates coverage of measles immunization varied based on the economic status, educational status, place of residence and subnational regions within the country. Coverage was higher for advantaged groups compared to disadvantaged groups. The poorest quintiles for all 4 years had 18.2% (2000), 24.8% (2005), 45.3% (2011) and 43.2% (2016) immunization coverage while the richest had 52.1% (2000), 52.5% (2005), 79.7% (2011), and 74.2% (2016) across the years (Fig. 1). This demonstrates a significant coverage difference between the two groups. Interestingly, coverage of measles immunization increased over the years from 2000 to 2016 for the poorest by 25 percentage points and richest by 22.1 percentage points on average (Fig. 1 and Table 1).

**Table 1:** Extent and trends of childhood measles immunization coverage across different socio-economic dimensions in Ethiopia from 2000 to 2016

Dimension of inequalities	Year							
	2000		2005		2011		2016	
	%(95%UI)	Pop n	%(95%UI)	Pop n	%(95%UI)	Pop n	%(95%UI)	Pop n
**Economic status**								
Quintile 1 (poorest)	18.22(13.91, 23.50)	457	24.89(19.86, 30.71)	450	45.30(38.25, 52.55)	441	43.22(35.86, 50.89)	504
Quintile 2	15.94(11.81, 21.15)	444	28.95(23.11, 35.59)	398	52.02(44.93, 59.03)	418	49.93(41.82, 58.05)	395
Quintile 3	22.31(16.74, 29.09)	464	37.62(31.60, 44.04)	381	51.97(42.85, 60.96)	394	54.40(46.42, 62.17)	449
Quintile 4	29.81(24.28, 36.00)	418	36.14(29.12, 43.81)	344	56.39(49.12, 63.38)	368	58.61(49.93, 66.79)	366
Quintile 5 (richest)	52.19(44.13, 60.13)	359	52.51(42.68, 62.15)	301	79.72(71.48, 86.05)	306	74.29(64.57, 82.09)	287
**Education**								
No education	22.07(19.06, 25.42)	1706	29.99(26.35, 33.89)	1456	49.88(45.03, 54.74)	1306	49.00(43.98, 54.04)	1257
Primary school	37.74(30.69, 45.35)	320	48.36(40.73, 56.08)	327	63.75(57.46, 69.60)	521	58.73(52.39, 64.79)	576
Secondary school or higher	61.65(46.62, 74.73)	118	63.38(48.10, 76.37)	92	89.40(77.00, 95.50)	101	78.82(64.08, 88.59)	170
**Residence**								
Rural	22.31(19.47, 25.43)	1919	32.24(28.84, 35.84)	1729	51.77(47.29, 56.23)	1656	51.50(47.12, 55.85)	1771
Urban	63.07(51.82, 73.07)	225	65.42(49.05, 78.80)	147	79.56(70.41, 86.42)	273	75.98(61.73, 86.12)	232
**Sex**								
Female	25.73(21.99, 29.86)	1036	33.23(28.94, 37.81)	917	55.71(50.39, 60.89)	919	55.78(49.95, 61.46)	1077
Male	27.40(23.68, 31.47)	1108	36.39(32.27, 40.73)	959	55.71(50.46, 60.85)	1009	52.65(47.42, 57.82)	926
**Region**								
Tigray	66.59(58.53, 73.77)	121	63.33(56.86, 69.35)	134	83.74(76.99, 88.80)	128	80.10(71.34, 86.68)	151
Affar	10.93(4.14, 25.88)	18	8.10(3.70, 16.80)	17	30.30(22.35, 39.64)	17	30.11(20.81, 41.41)	20
Amhara	27.06(21.13, 33.94)	564	34.75(28.72, 41.32)	482	61.96(53.02, 70.16)	445	61.91(52.04, 70.88)	364
Oromiya	19.60(15.78, 24.07)	904	29.40(22.51, 37.37)	691	45.86(38.82, 53.07)	810	43.20(36.24, 50.43)	880
Somali	39.29(16.36, 68.17)	24	6.36(2.05, 18.03)	78	39.49(27.28, 53.16)	50	48.14(36.43, 60.06)	75
Ben-gumz	19.31(12.17, 29.24)	18	33.37(22.22, 46.75)	16	67.21(57.78, 75.43)	22	70.78(60.89, 79.03)	20
SNNP	24.30(18.34, 31.44)	442	37.69(32.04, 43.70)	407	57.80(50.14, 65.11)	390	57.63(49.46, 65.39)	418
Gambela	20.00(10.51, 34.73)	5	30.74(17.09, 48.87)	5	51.72(41.12, 62.17)	7	62.14(50.57, 72.48)	5
Harari	58.58(47.33, 69.01)	4	39.94(31.64, 48.86)	4	64.72(52.25, 75.47)	5	53.60(43.56, 63.35)	4
Addis	88.27(80.37, 93.25)	33	78.75(65.80, 87.71)	31	93.46(85.79, 97.12)	42	93.11(86.30, 96.66)	52
Dire dawa	52.52(40.49, 64.27)	7	55.72(46.00, 65.02749	7	79.89(70.93, 86.61)	6	86.87(80.14, 91.55)	9
National measles coverage	26.59		34.85		55.71		54.33

**Fig. 2:**
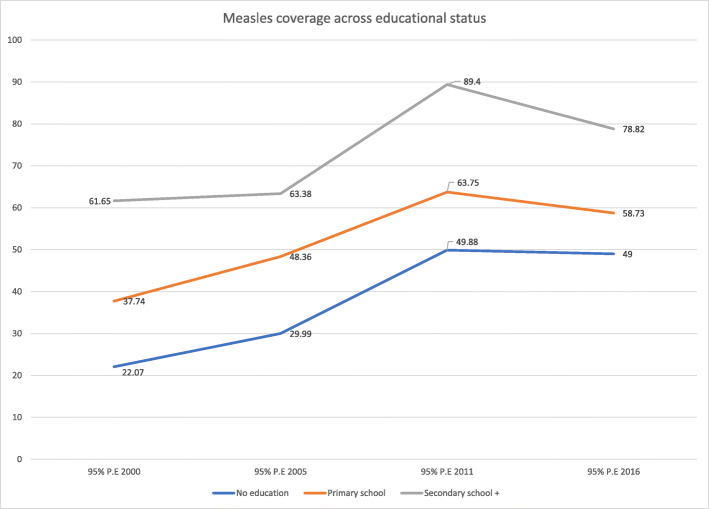
Coverage and trends of childhood measles immunization based on educational status in Ethiopia from 2000 to 2016

Coverage of measles immunization varied based on educational status with higher coverage among the more educated as compared to the non-educated participants. This is seen in 2016’s 30 percentage point difference on average in measles immunization coverage between participants with secondary or higher education and those with no education. The coverage across all educational categories increased from 2000 to 2011. Then, from 2011 to 2016, it leveled off for non-educated and primary educated groups but decreased slightly for individuals with secondary and higher education. There was a general increasing trend for measles immunization coverage for the education categories across time (2000–2016 (Fig. 2 and Table 1).

**Fig. 3:**
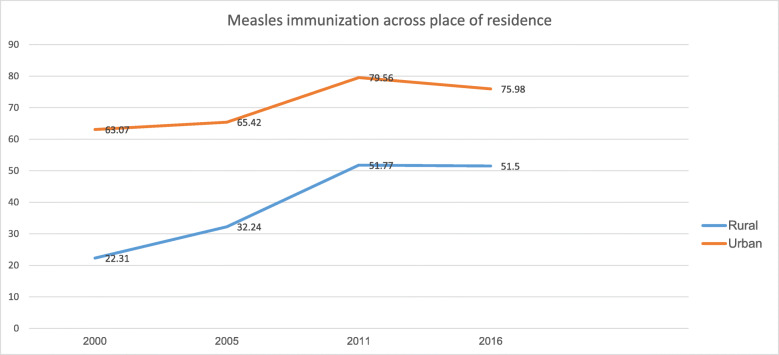
Coverage and trends of childhood measles immunization based on place of residence in Ethiopia from 2000 to 2016

Figure 3 illustrates a significant difference in measles immunization coverage between rural and urban residents in Ethiopia across all four time periods. Coverage for rural residents increased by, on average, more than 29 percentage points from 2000 to 2016. However, the increase in coverage only occurred from 2000 to 2011 for urban residents by a 16.5 percentage points and leveled off between 2011 and 2016. See Table 1 for details.

**Fig. 4:**
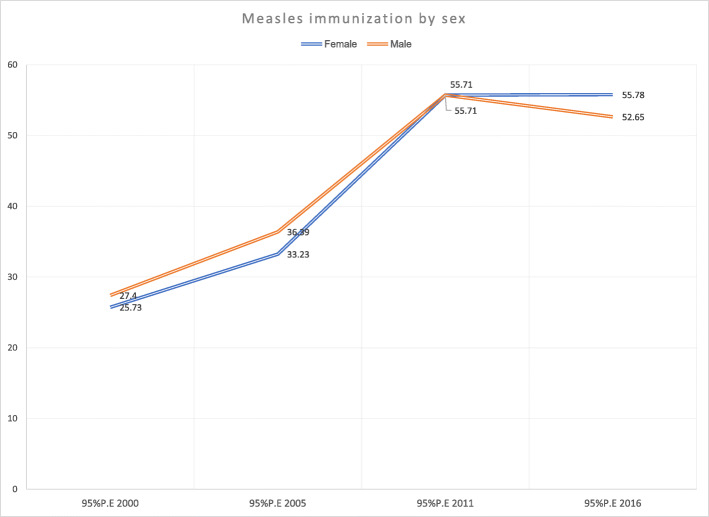
Coverage and trends of childhood measles immunization based on child sex in Ethiopia from 2000 to 2016

Findings show there was no sex-based difference in childhood measles immunization in Ethiopia across all years. The coverage pattern for both male and female children increased from 2000 to 2011 and were level between 2011 to 2016 (Fig. 4 and Table 1).

Significant coverage difference in childhood measles immunization existed in the studied regions in Ethiopia across all four rounds. The most recent 2016 survey revealed a 63 percentage point average difference in measles immunization coverage between the region with the lowest coverage (Afar) and that with the highest coverage (Addis Ababa). The pattern of immunization coverage across all studied regions fluctuated over the 16 years; some regions increased by 51.4 percentage points (Ben-Gumuz), while others decreased by 4.9 percentage points (Harari) on average (Table 1).

### Magnitude and trends of inequality

**Fig. 5:**
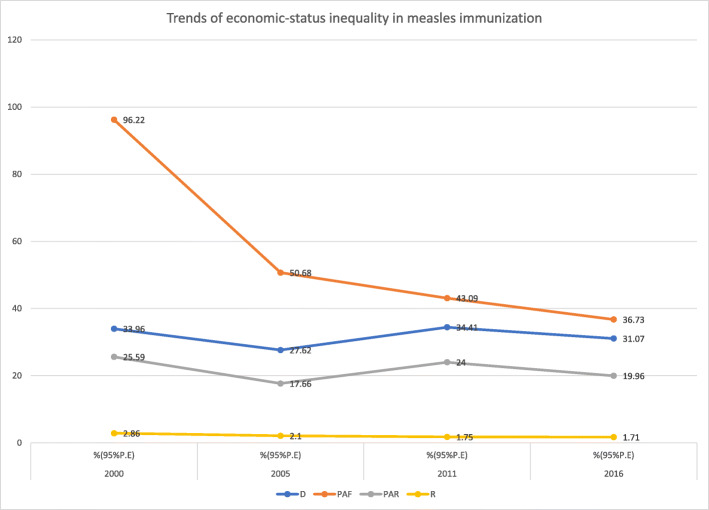
Trends of economic status inequality in childhood measles immunization in Ethiopia from 2000 to 2016

Findings from both absolute (D, PAR) and relative (R, PAF) summary measures revealed economic inequality in measles immunization in Ethiopia from 2000 to 2016 (Table 2). The simple measures indicated the pattern of absolute (D) and relative (R) inequality were consistent between 2000 and 2016. However, the complex absolute measure (PAR) showed a decreasing pattern of measles coverage inequality between 2000 to 2005, followed by an increase from 2005 to 2011 which continued to level off in 2016. Similarly, the complex relative measure (PAF) indicated a substantial decrease in inequality from 2000 to 2005 which remained constant over subsequent survey years (Fig. 5).

**Table 2:** Magnitude and trends of socio-economic inequality in measles immunization in Ethiopia from 2000 to 2016

Dimension	Year				
	Measure	2000	2005	2011	2016
		%(95%UI)	%(95%UI)	%(95%UI)	%(95%UI)
**Economic status**	D	33.96(24.62, 43.30)	27.62(16.42, 38.82)	34.41(24.20, 44.63)	31.07(19.48, 42.66)
	PAF	96.22(83.97, 108.46)	50.68(40.36, 61.00)	43.09(35.72, 50.45)	36.73(29.78, 43.68)
	PAR	25.59(22.33, 28.84)	17.66(14.06, 21.26)	24.00(19.90, 28.11)	19.96(16.18, 23.73)
	R	2.86(1.99, 3.73)	2.10(1.50, 2.71)	1.75(1.43, 2.08)	1.71(1.35, 2.08)
**Education**	D	39.57(24.88, 54.26)	33.39(18.48, 48.30)	39.52(29.52, 49.51)	29.82(16.57, 43.06)
	PAF	131.77(127.71, 135.84)	81.87(78.26, 85.48)	60.46(57.70, 63.23)	45.07(41.95, 48.18)
	PAR	35.05(33.97, 36.13)	28.53(27.27, 29.79)	33.69(32.14, 35.23)	24.49(22.79, 26.18)
	R	2.79(2.029, 3.55)	2.11(1.56, 2.66)	1.79(1.54, 2.03)	1.60(1.30, 1.90)
**Place of residence**	D	40.76(29.64, 51.88)	33.17(17.58, 48.77)	27.78(18.63, 36.93)	24.48(11.4637.50)
	PAF	137.14(133.95, 140.34)	87.72(85.58, 89.86)	42.80(41.20, 44.40)	39.84(38.40, 41.27)
	PAR	36.48(35.63, 37.32)	30.57(29.82, 31.31)	23.84(22.95, 24.74)	21.64(20.86, 22.43)
	R	2.82(2.21, 3.43)	2.02(1.50, 2.54)	1.53(1.33, 1.74)	1.47(1.20, 1.74)
**Sex**	D	−1.67(−7.18, 3.83)	−3.16(−9.27, 2.94)	−0.0(− 7.40, 7.38)	3.13(−4.64, 10.90)
	PAF	0(−6.79, 6.79)	0(−6.04, 6.04)	0(−3.79, 3.79)	2.66(−1.67, 6.99)
	PAR	0(−1.80, 1.80)	0(−2.10, 2.10)	0(− 2.11, 2.11)	1.44(−0.90, 3.80)
	R	0.93(0.74, 1.13)	0.91(0.75, 1.07)	0.99(0.86, 1.13)	1.05(0.90, 1.21)
**Region**	D	77.33(65.41, 89.24)	72.39(59.44, 85.33)	63.15(52.99, 73.30)	62.99(51.51, 74.47)
	PAF	231.85(162.15, 301.55)	125.96(108.61, 143.31)	67.74(26.32, 109.17)	71.35(31.76, 110.95)
	PAR	61.67(43.13, 80.21)	43.90(37.85, 49.94)	37.74(14.66, 60.82)	38.77(17.26, 60.28)
	R	8.06(0.57, 15.56)	12.37(−1.28, 26.03)	3.08(2.18, 3.98)	3.09(2.01, 4.17)

**Fig. 6:**
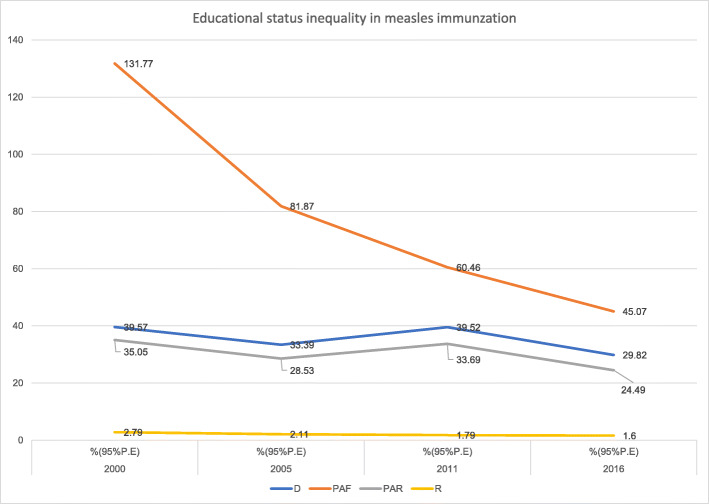
Trends of educational status inequality in childhood measles immunization in Ethiopia from 2000 to 2016

Like economic status inequality, inequality in measles immunization coverage existed based on education status in Ethiopia from 2000 to 2016 (Table 2). While the absolute (D) educational status inequality was consistent from 2000 to 2016, the relative (R) educational inequality decreased substantially between the first and the last surveys. However, the complex absolute measure (PAR) fluctuated; inequality decreased from 2000 to 2005, increased between 2005 to 2011, and again decreased between the last two survey years. Interestingly, (PAF) revealed a consistently decreasing trend in educational status inequality from 2000 to 2016 (Fig. 6).

**Fig. 7:**
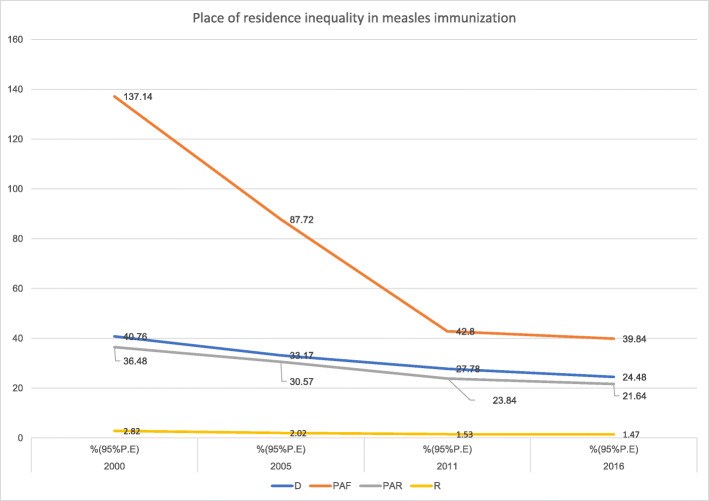
Trends of place of residence inequality in childhood measles immunization in Ethiopia from 2000 to 2016

Inequality in measles immunization existed based on place of residence in Ethiopia from 2000 to 2016. Despite a slight overlap of UIs, the simple measures revealed a decreasing pattern of inequality. Both the complex measures showed a decreasing pattern of residence inequality in the country from 2000 to 2016 (Fig. 7).

**Fig. 8:**
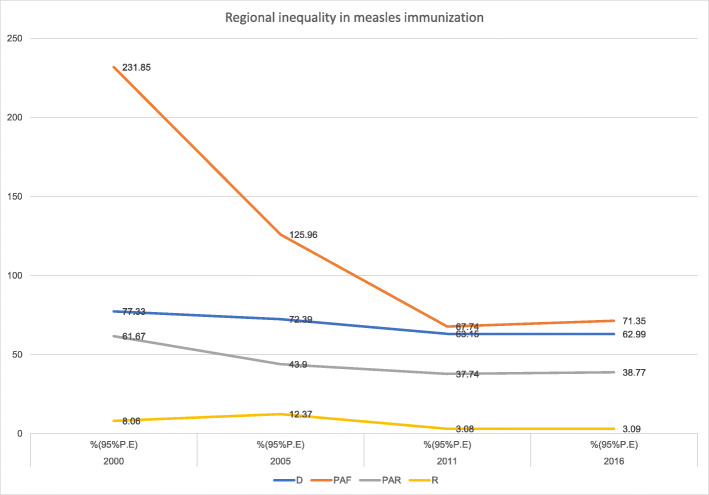
Trends of regional inequality in childhood measles immunization in Ethiopia from 2000 to 2016

Interestingly, a general pattern of regional inequality existed in Ethiopia across all the years using all the measures except for the simple measure (R) in 2000 and 2005. The complex relative measure (PAF) decreased from 2000 to 2011 and leveled off between 2011 to 2016. Even with little overlap in the Uncertainty Intervals (UIs), absolute complex measure (PAR) suggested evidence of decreasing regional inequality from 2000 to 2011 which then remained constant between 2011 and 2016 (Fig. 8).

Table 2 shows no absolute (D, PAR) or relative (R, PAF) sex inequality in measles immunization coverage in Ethiopia between 2000 and 2016.

## Discussion

Using the Ethiopia Demographic Health and Survey data, the magnitude and trends of socio-economic, sex related and location-based inequality in childhood measles immunization coverage were investigated between 2000 and 2016. A general over-time increase was evident for the national measles immunization coverage during the first three surveys, which leveled off by the last survey in 2016. Overall national levels of measles immunization showed a median increase of 1.73% per year (Table 1). Interestingly, there was a larger increase in the poorest quintile (1.56 per year) as compared to the richest quintile (1.38 per year).

In Ethiopia, a pronounced level of economic inequality was observed between all the rungs of economic status subgroups. There was a staggering 31 percentage points difference between the poorest and richest quintiles in 2016. The 2016 Population Attributable Factor (PAF) was 36.7%; this indicates that if the coverage of measles immunization in the four quintiles was the same as the richest wealth quintile, the national measles immunization coverage would be improved by 36.7% from the current state. The simple measures in that same year indicated that coverage of measles immunization in the richest subgroup was 1.7 times higher than the poorest subgroup. Household income can play a substantial role in access to care; numerous unintended expenses related to immunizations (i.e. transportation to clinics) may be tolerable in the richest families but not possible for the poorest ones [41].

This study showed absolute and relative educational status inequality in measles immunization in Ethiopia from 2000 to 2016. Based on the point estimates, in the most recent survey, the simple measures showed a Difference of 29.8 percentage points in coverage between the non-educated and most educated households while the Ratio showed the more educated mothers were 1.6 times more likely to have coverage for their children. The PAF value in the 2016 survey showed that if measles immunization coverage in all subgroups were at the same level as household with mothers who have secondary school education or higher, the overall country’s measles immunization coverage could be improved by a staggering 45%. Similar findings were reported by other studies in Ethiopia [42], Democratic Republic of Congo [43] and Nigeria [44]. Educated mothers are more likely to use health care services (i.e. childhood immunization) because of enhanced access and understanding to relevant healthcare information [45, 46]. Furthermore, educated mothers tend to have better communication skills which facilitate interactions with health workers, leading to a better understanding of immunization schedules and practices [45–47].

Findings from this study showed no sex inequality in measles immunization coverage as there was no variation between male and female children. However, there was inequality when comparing the pattern of change in measles immunization coverage in rural and urban areas. In 2016, urban regions were 24.5 percentage points higher in measles coverage than rural areas. Similarly, in the earlier survey years, urban regions had more coverage, however over time, rural regions seemed to greatly increase their immunization coverage and urban areas slowly increased but then declined in 2016. Despite this, residence related inequities in coverage remained in Ethiopia, with the rural setting falling behind the urban area in all the studied years. The PAF value in 2016 indicated that without the existence of inequality between urban and rural, coverage of measles immunization in the country could be improved by close to 40%. The pro-urban finding around this vaccine is not surprising as utilization of health services in rural regions is inadequate because of availability, accessibility, quality of services and the characteristics of the users and the communities in which they live. Geographical access is a key factor in utilization of healthcare services as predominantly seen in rural areas with limited delivery of healthcare services [48, 49].

Another main finding from our study is presence of significant absolute and relative regional inequality in Ethiopia in all survey rounds. There was significantly lower measles immunization coverage in some regions as compared to other regions within the country. In 2016, the difference in immunization coverage between Addis Ababa (region with highest coverage) and Afar (region with lowest coverage) was 63 percentage points. To further illustrate the regional inequality, the ratio measure showed Addis Ababa had three times the immunization coverage compared to Afar. If all the other regions had the same measles immunization coverage level as that of Addis Ababa in 2016, the overall national coverage of measles immunization would be improved by 39 percentage points on average, as indicated by PAR. This result is consistent with a previous study in Ethiopia that showed measles immunization varied across different regions with in the country [50]. Culture, religion, economic status, vaccine supply, availability and accessibility of immunization health services could account for regional differences.

Our study has some strengths. This study made use of nationally-representative surveys to explore the nature and degree of inequality in Ethiopia from different perspectives. In the current literature, studies on this topic are rare and do not show significance. From this perspective, we believe the present findings make an important contribution to the knowledge base and provide a solid basis for further research. However, this paper has several limitations. First, the DHS data is cross-sectional and therefore no causality can be inferred from the associations. The authors have no influence over the selection and measurement of the variables when using secondary database analysis Second, we were not able to examine the effect of determinant factors on the observed measles inequality. Future studies might employ a decomposition technique to understand the individual contribution of different factors on measles inequality.

## Conclusions

Several key findings from this study emerged. First, the progress in Ethiopia’s national coverage of measles immunizations increased until 2011, after which it plateaued though the absence of UIs around point estimates makes it difficult to comment on the progress. Second, significant inequality in childhood measles immunization was seen in subgroups based on economic status, educational status, place of residence and region in the country. Even though the patterns of inequality were different in the relative and absolute inequality measures used, inequalities were observed, leading to the need for multilevel interventions to reach missed children. Political determination is vital to progress and the reduction of such inequalities in Ethiopia. Where inequalities exist, the national, regional and district levels of government should make a pledge to reduce inequalities in coverage of measles immunization, formulating equity-sensitive strategies and assigning sufficient human and financial resources to implement them based on scope. International and national immunization experts and researchers need to continue investigating and monitoring inequalities related to measles immunization and propose possible solutions to achieve the related sustainable development goals.

## Data Availability

The datasets generated and/or analyzed during the current study are available in the WHO’s HEAT version 3.1 [https://www.who.int/gho/health_equity/assessment_toolkit/en/].

## Abbreviations

D: Difference
DHS: Demographic and Health Survey
EA: Enumeration Area
EDHS: Ethiopia Demographic and Health Survey
HEAT: Health Equity Assessment Toolkit
PAR: Population Attributable Risk
R: Ratio
SDG: Sustainable Development Goal
UI: Uncertainty Interval
WHO: World Health Organization
